# SARS-CoV-2 mRNA vaccine BNT162b2 triggers a consistent cross-variant humoral and cellular response

**DOI:** 10.1080/22221751.2021.2004866

**Published:** 2021-12-01

**Authors:** D. Mileto, C. Fenizia, M. Cutrera, G. Gagliardi, A. Gigantiello, A. De Silvestri, A. Rizzo, A. Mancon, M. Bianchi, F. De Poli, M. Cuomo, I. Burgo, M. Longo, S. G. Rimoldi, C. Pagani, S. Grosso, V. Micheli, G. Rizzardini, R. Grande, M. Biasin, M. R. Gismondo, A. Lombardi

**Affiliations:** a Laboratory of Clinical Microbiology, Virology and Bioemergencies, ASST Fatebenefratelli Sacco, L. Sacco University Hospital, Milan, Italy; b Department of Biomedical and Clinical Sciences “L. Sacco”, University of Milan, Milan, Italy; c Department of Pathophysiology and Transplantation, University of Milan, Milan, Italy; d Clinical Epidemiology and Biometeric Unit, Fondazione IRCCS Policlinico San Matteo, Pavia, Italy; e Hematology and Transfusion Medicine, ASST Fatebenefratelli Sacco, L. Sacco University Hospital, Milan; f Division of Infectious Diseases, ASST Fatebenefratelli Sacco, L. Sacco University Hospital, Milan, Italy

**Keywords:** SARS-CoV-2 bnt162b2 mRNA vaccine, Humoral response, T-cell mediated respone, SARS-CoV-2 variants of concern

## Abstract

As the SARS-CoV-2 pandemic continues to rage worldwide, the emergence of numerous variants of concern (VOC) represents a challenge for the vaccinal protective efficacy and the reliability of commercially available high-throughput immunoassays. Our study demonstrates the administration of two doses of the BNT162b2 vaccine that elicited a robust SARS-CoV-2-specific immune response which was assessed up to 3 months after full vaccination in a cohort of 37 health care workers (HCWs). SARS-CoV-2-specific antibody response, evaluated by four commercially available chemiluminescence immunoassays (CLIA), was qualitatively consistent with the results provided by the gold-standard *in vitro* neutralization assay (NTA). However, we could not observe a correlation between the quantity of the antibody detected by CLIA assays and their neutralizing activity tested by NTA. Almost all subjects developed a SARS-CoV-2-specific T-cell response. Moreover, vaccinated HCWs developed a similar protective neutralizing antibodies response against the EU (B.1), Alpha (B.1.1.7), Gamma (P.1), and Eta (B.1.525) SARS-CoV-2 variants, while Beta (B.1.351) and Delta (B.1.617.2) strains displayed a consistent partial immune evasion. These results underline the importance of a solid vaccine-elicited immune response and a robust antibody titre. We believe that these relevant results should be taken into consideration in the definition of future vaccinal strategies.

## Introduction

At the end of 2019, a new coronavirus, SARS-CoV-2, was first described in Wuhan, China, as being responsible for pneumonia within the scenario of a new disease: coronavirus disease 2019 (COVID-19). To contain the COVID-19 spread, countries worldwide have adopted several long-lasting rigorous restrictions but the dramatic and unpredictable health, social, and economic consequences undermine the compliance to further stringent lockdowns. The acquisition of immunity by vaccination represents, therefore, the most promising chance to contain the COVID-19 pandemic.

The fine-tuning of COVID-19 vaccines has been extraordinarily rapid and timely, and has been demonstrated to be safe and largely proficient in controlling symptomatic SARS-CoV-2 infections; however, the precise kinetic of SARS-CoV-2-specific immune responses, elicited by vaccination, needs to be further addressed. Data produced so far suggest that virus-specific neutralizing antibodies targeting the receptor binding domain (RBD) of the spike (S) protein decay to some extent over a period of several months [1]. Nonetheless, they remain detectable [2] and nearly all vaccinated, re-infected, or breakthrough-infected subjects show mild symptoms, suggesting that vaccine-driven immune memory results in protection from severe COVID-19.

The appearance of SARS-CoV-2 strains displaying mutations in the different spike protein domains raises some worries on the possibility of evading vaccination-induced neutralizing antibodies. These variants include the B.1.1.7 (α) strains first detected in the UK and now spread worldwide; the B.1.351 (β) and P.1 (γ) lineages identified in South Africa and Brazil, respectively; the B.1.525 first identified in Nigeria (η); and the B.1.617.2 strain recently isolated in India (δ). The Alpha strain developed a non-synonymous mutation at amino acid 501 (N501Y); the Beta and Gamma variants additionally acquired amino acid replacements at 417 and 484 positions (K417N/T, E484K), among others, shared by the Eta variant as well; while the Indian Delta variant overall displays the following mutations: T19R, 156del, 157del, R158G, L452R, T478K, D614G, P681R, and D950N [3]. Besides, several mutations raised in the N-terminal domain (NTD) of these lineages, implicating an *in vivo* selective pressure on the RBD and NTD sites. The appearance of these variants, with potential lowered susceptibility to antibody responses, could challenge the effectiveness of the worldwide vaccinal campaign, as already documented in the scientific literature [4–13].

Likely, alongside humoral immunity, vaccine-induced immunological memory relies also on the induction of cellular immunity. This is driven by CD4^+^ and CD8^+^ T-cells, which employ different protective strategies contributing to the control of SARS-CoV-2. Studies documented a protective T-cell response in patients with COVID-19 and the reported presence of SARS-CoV-2-specific T-cell reactivity in uninfected subjects raise remarkable queries concerning cross-reactivity due to previous infections with other coronaviruses. Wide-ranging research works on the cellular immune response to vaccination and its duration are currently under investigation as well as its role in cross-protection to the new emerging viral variants.

Herein, we assessed the humoral immune response against SARS-CoV-2 European strain (lineage B.1) on serum from 37 BNT162b2 mRNA-vaccinated health care workers (HCWs), who were never infected by SARS-CoV-2. For each subject, enrolled analyses were performed over a 3-month span of time from the second vaccine dose. At each time point, results obtained employing four different chemiluminescence immunoassays (CLIA) were compared to those derived by gold-standard virus neutralization test requiring live pathogen [14]. At T5 (30 days after dose II), collected serum samples were tested even against Alpha, Beta, Gamma, Delta, and Eta variants, while T-cell response was assessed by QuantiFERON assay at T6 (90 days after dose II).

## Materials and methods

### Study design

**Figure 1.**
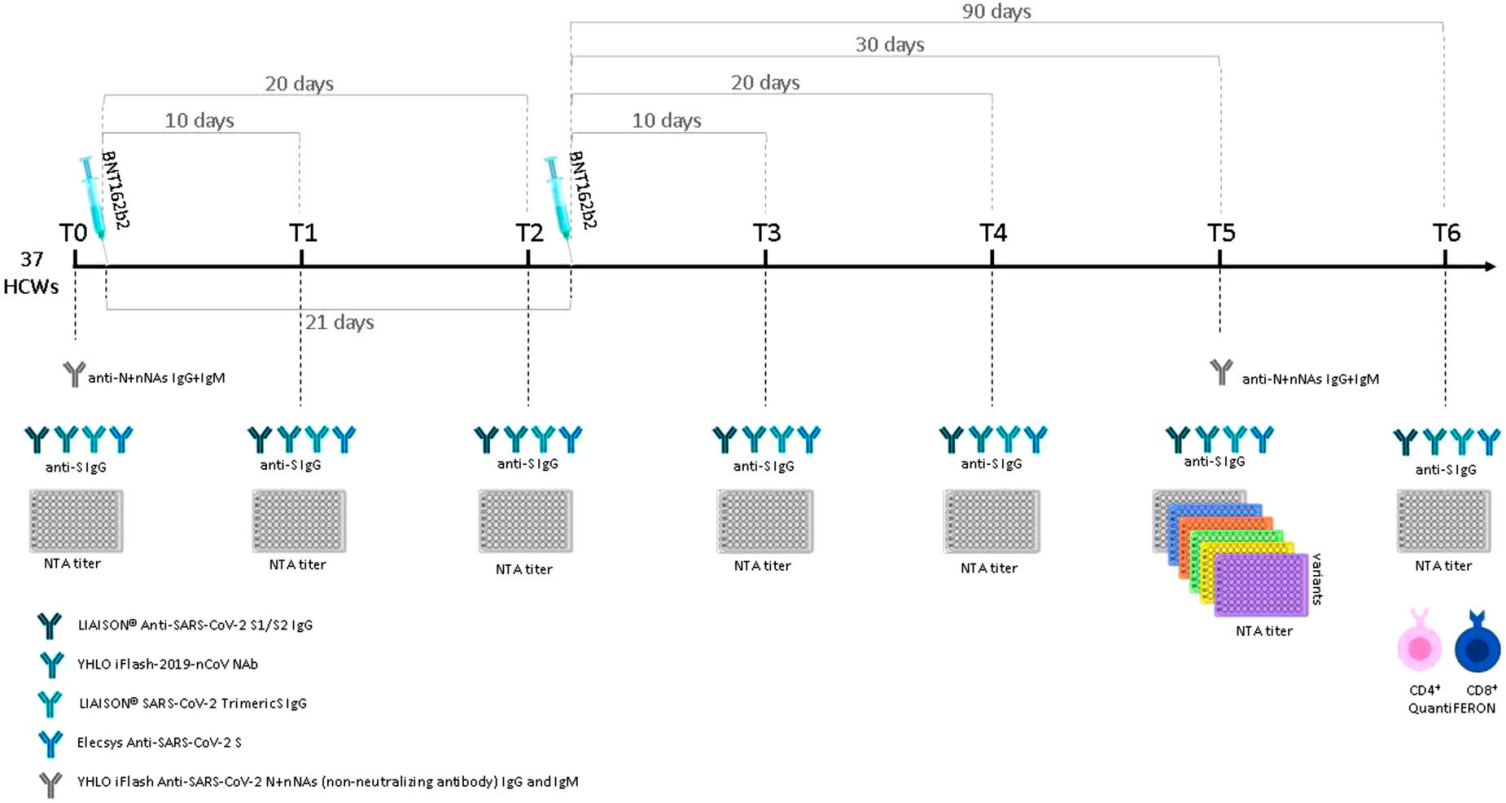
Synoptic representation of the study design with timing and type of analyses.

An observational, longitudinal prospective study was designed to evaluate the development of immune response in infection-naive HCWs induced by BNT162b2 (Comirnaty) anti-SARS-CoV-2 vaccine; vaccine was administered according to the Pfizer vaccination schedule: dose II administered 21 days after dose I. The primary end-point of the study was to characterize the development of SARS-CoV-2-specific neutralizing antibodies, monitoring immunoglobulin kinetic at the following consecutive time-points: one day before vaccination (T0), 10 days after dose I (T1), 20 days after dose I (T2), 10 days after dose II (T3), 20 days after dose II (T4), 30 days after dose II (T5), and 90 days after dose II (T6); evaluation was conducted by analyzing the subjects’ serum samples with four different CLIA. Secondary end-points were as follows: (i) evaluation of effective neutralization against main relevant SARS-CoV-2 variants; (ii) agreement among different CLIA assays and the gold-standard neutralization assay (NTA); (iii) assessment of specific T-cell response against viral S protein by means of Interferon-γ Release Assay (IGRA) at T6. The study design is summarized in Figure 1.

### SARS-CoV-2 Ab measurement

All collected serum samples were analyzed using four different CLIA assays: LIAISON^®^ SARS-CoV-2 S1/S2 IgG (311450 – DiaSorin, Saluggia, Italy) to measure the antibodies against the SARS-CoV-2-native S1/S2 proteins, while iFlash-2019-nCoV Nab (C86109 – Shenzhen YHLO Biotech Co, Shenzhen, China), LIAISON^®^ SARS-CoV-2 TrimericS IgG (P/N311510 – DiaSorin, Saluggia, Italy), and Elecsys Anti-SARS-CoV-2 S (09 289 267 190 – Roche Diagnostics Rotkreuz, Switzerland) to quantify the specific RBD-binding antibodies. Moreover, samples collected at T0 and T5 were analyzed using iFlash SARS-CoV-2 IgG and IgM (C86095G – C86095M – Shenzhen YHLO Biotech Co, Shenzhen, China) to exclude a possible ongoing asymptomatic infection since that the assay targets nucleocapside and spike proteins (non-neutralizing antibodies). All the assay characteristics are summarized in Table 1.

NTA was used to quantify the titre of neutralizing antibody for all samples at each time point using the SARS-CoV-2 lineage B.1 (EU): results were considered positive if higher or equal to 1:10 serum titre [15,16].

Furthermore, the serum samples collected at 1 month after dose II (T5) were used to evaluate the *in vitro* neutralizing response against the different isolated variants. Further details are reported in Supporting Information.

### SARS-CoV-2 variants

SARS-CoV-2 variants, including the lineage B.1 (EU) (accession number: EPI_ISL_412973), assumed as comparator virus, as well as Alpha (lineage B.1.1.7) (accession number: EPI_ISL_1909218), Beta (lineage B.1.351) (accession number: EPI_ISL_1578464), Gamma (lineage P.1) (accession number: EPI_ISL_1578455), Delta (lineage B.1.617.2) (accession number: EPI_ISL_1970729), and Eta (lineage B.1.525) (accession number: EPI_ISL_1649798) were isolated from positive nasopharyngeal swabs (NPS). Viruses were isolated, propagated, and titrated to prepare themfor neutralization assay using the permissive cell line VERO C1008 (Vero 76, clone E6, Vero E6; ATCC1CRL-1586TM). All the strains were identified by means of whole genome sequencing and the sequences were submitted to GISAID.

### T-cell response evaluation

The T-cell response induced by vaccination was evaluated at T6 by a whole-blood IGRA using QuantiFERON^®^ (QNF) ELISA SARS-CoV-2 assay (QIAGEN, Hilden, Germany).

Briefly, venous blood sample was collected in four dedicated Quantiferon^®^ tubes, namely, Ag1 for CD4^+^ T cell stimulation, Ag2 for CD4^+^ plus CD8^+^ T cell stimulation, Mitogen as positive control, and Nil as negative control. Both Ag1 and Ag2 were coated with a mixture of S protein peptides to induce IFN-γ production. After a 16–24 h incubation at 37°C, tubes were centrifuged, and the separated plasma was analyzed by a microplate of enzyme-linked immunosorbent assay (ELISA) for IFN-γ dosage: a value of 0.15 IU/ml was used as the positive cut-off. The QNF ELISA is for research use only.

Twenty unvaccinated healthy controls with no nucleocapside and spike antibodies were used as controls (HCs).

### Statistical analysis

In descriptive statistics, data mean and standard deviation (sd) and median and range were used for quantitative variables with Gaussian and non-Gaussian distribution, respectively, as well as numbers and percentages for qualitative variables. Ratios between NTA and NTA of variants were described as geometric mean. Quantitative variables association was evaluated through Pearson correlation coefficient *r*. The intraclass correlation coefficient (ICC) among NTA and Ab measurements (or positivity) was calculated after fitting mixed models (with patients and time as random factors) to take into account the clustered nature of the data. Assay’s sensitivity and specificity were calculated using NTA as gold-standard. Statistical analyses were performed by STATA software (StataCorp LLC, Texas, USA) and MedCalc (MedCalc Software Ltd, Ostend, Belgium). Differences were considered significant when *P* value < 0.05.

## Results

A total of 37 SARS-CoV-2 infection-naive HCWs vaccinated between 28 December 2020 and 23 February 2021 was enrolled. Among them, nine were males and 28 were females with a median age of 40 (range 24–65) years. All the enrolled HCWs were tested for the presence of non-neutralizing anti-SARS-CoV-2 N and S Subunits (NNAs), to check for previous SARS-CoV-2 infection. None of them resulted to have SARS-CoV-2 N and S antigens experienced neither at T0 nor T5 (supporting information).

### Antibody response in SARS-CoV-2-vaccinated HCWs

**Figure 2.**
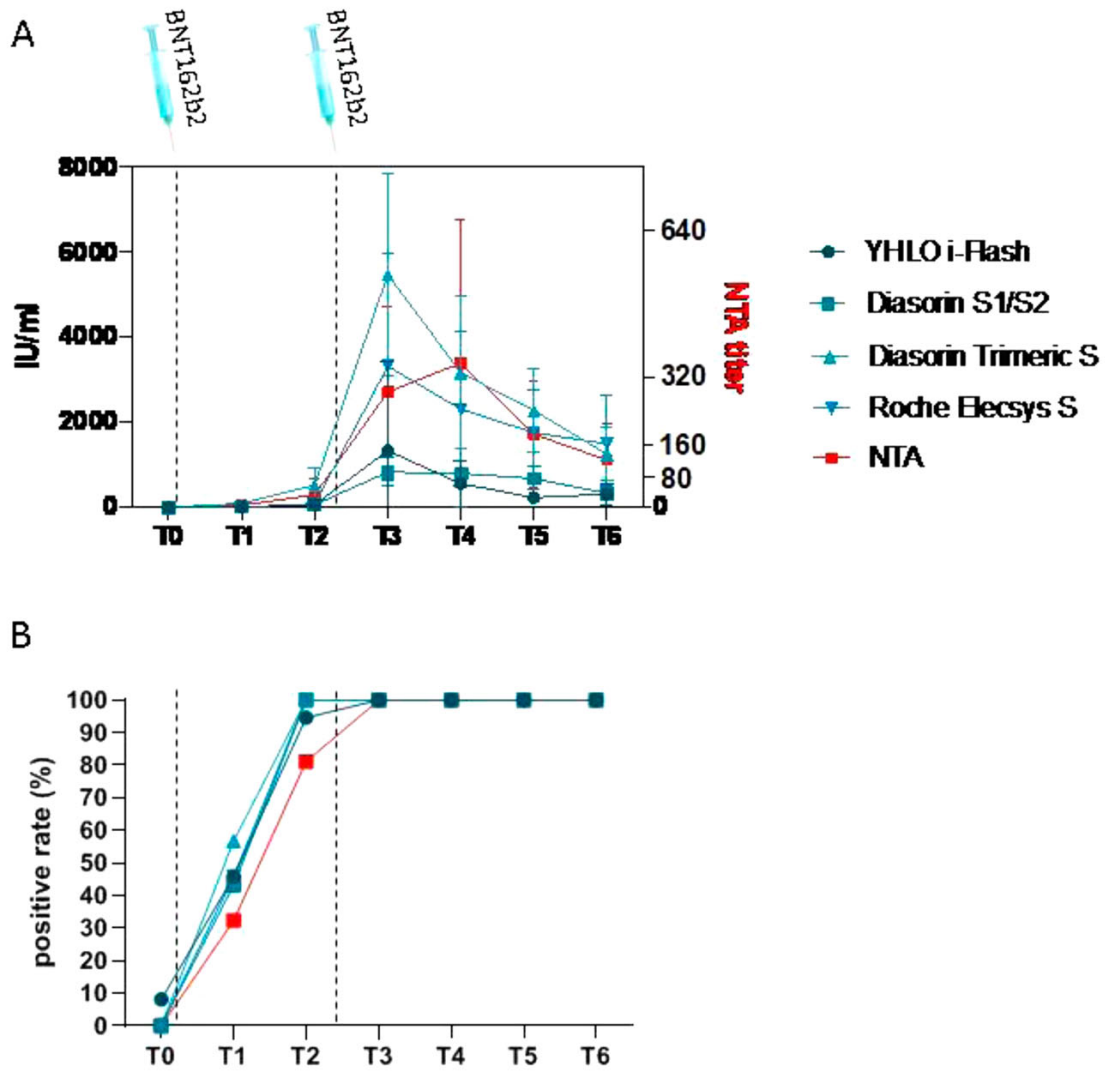
Humoral response. Panel (A) Anti-SARS-CoV-2 specific antibody titre overtime, quantified by four different CLIA methods and by neutralization assay (NTA). Panel (B) Percentage of subjects detected positive to anti-SARS-CoV-2 specific antibodies overtime, quantified by four different CLIA methods and by neutralization assay (NTA). Time of vaccinations is represented by vertical dashed lines.

Results of the kinetics of systemic humoral response elicited by vaccine showed that a weak production of SARS-CoV-2-specific antibodies (Figure 2(A)) was detectable in nearly 50% of the enrolled HCWs since 10 days post the first vaccine dose (T1) (Figure 2(B)). The percentage of subjects resulting positive to the antibodies increased at T2 above 90% and stabilized at 100% for the following timepoints (Figure 2(B)). The antibody titre reached the climax 10 days after the second dose (T3), then to gradually decrease over time (Figure 2(A)).

A similar trend was observed for neutralizing antibodies analyzed by NTA: 12/37 (32.4%) HCWs developed detectable SARS-CoV-2 NT antibodies at T1 (mean ± sd: 5.1 ± 13.46), and neutralizing antibody titres increased to 81.1% at T2 (mean ± sd: 30.3 ± 38.19), becoming detectable in all but seven subjects (Figure 2(B)). However, neutralization antibody peak production was registered slightly later, at T4 (mean ± sd: 339.5 ± 337.75) (Figure 2(A)). Overall, there was good qualitative agreement between IgG quantification by CLIA assays and NTA (Diasorin S1/S2: ICC = 0.85; i-Flash: ICC = 0.78; Roche Elecsys S: ICC = 0.81; Diasorin Trimeric: ICC = 0.81), but quantitative correlation was almost absent taking into account all the timepoints (Diasorin S1/S2: ICC = 0.51; i-Flash: ICC = 0.18; Roche Elecsys S: ICC = 0.13; Diasorin Trimeric: ICC = 0.11). No correlation with sex or age was detected with antibody production quantified by different techniques at any timepoint.

### T-cell response in SARS-CoV-2-vaccinated HCWs

QuantiFERON assay was used for detecting CD4^+^ and CD4^+^ plus CD8^+^ SARS-CoV-2-specific T-cell-mediated response in vaccinated subjects at T6.

**Figure 3.**
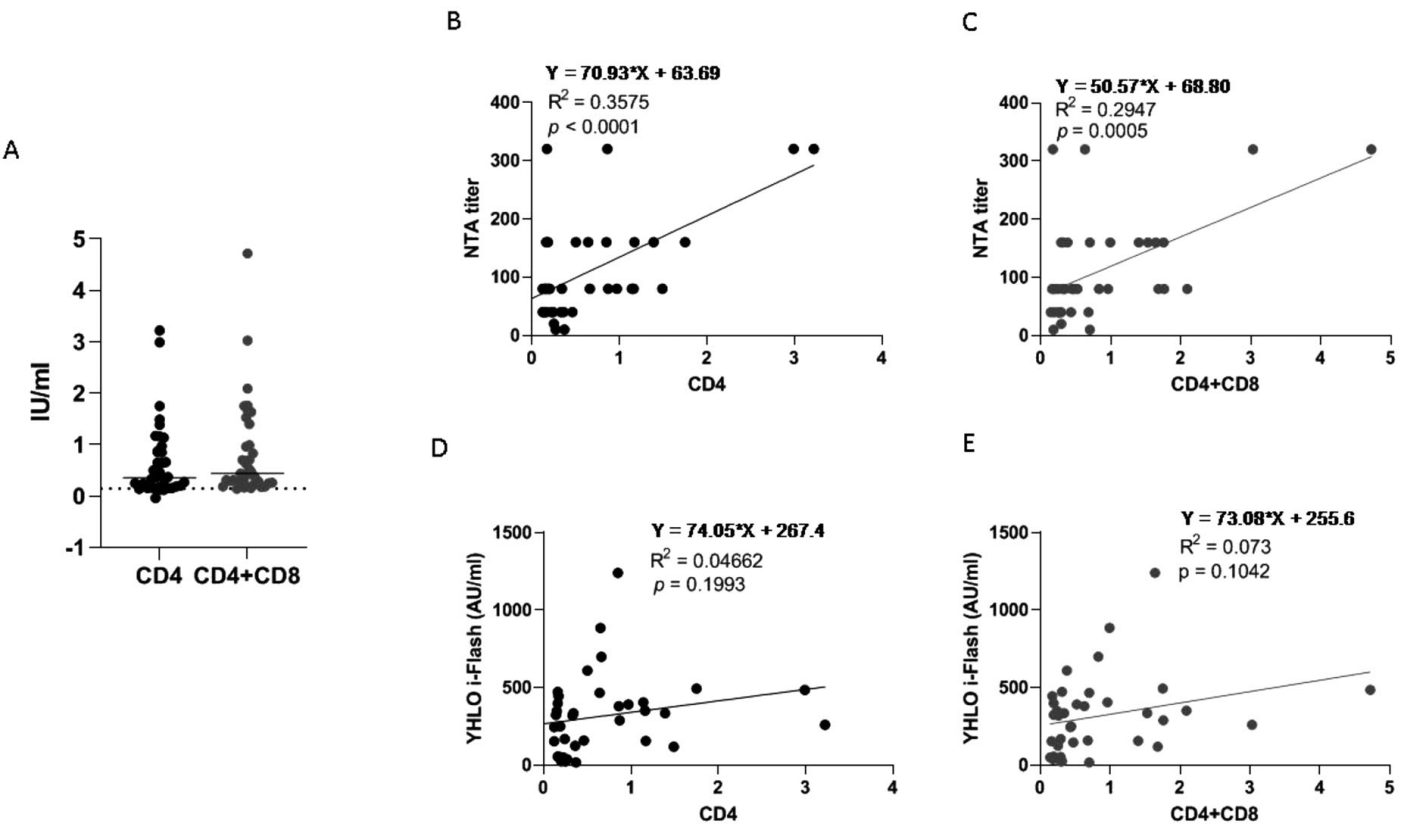
Cellular response. Panel (A) T-cell mediated response measured by QuantiFERON assay related to CD4+ T-cell only (black) or CD4+ and CD8+ T-cells together (grey). Data refer to T6 and are expressed as international unit per millilitre (IU/ml); the cut-off is 0.15 IU/ml. Panel (B and C) correlation between the neutralization assay (NTA) and cellular CD4+ and CD4++CD8+ T-cells response, respectively. Panel (D and E) correlation between anti-SARS-CoV-2 specific antibodies measured by YHLO i-Flash and cellular CD4+ and CD4++CD8+ T-cells response, respectively. The correlation with the quantification of anti-SARS-CoV-2 specific antibodies measured by the other methods is depicted in figure S2.

All but four SARS-CoV-2-vaccinated subjects (33/37, 89.2%) showed SARS-CoV-2-specific CD4^+^ T-cell specific response at T6. Whereas CD4^+^ plus CD8^+^ SARS-CoV-2-specific cell-mediated immunity was observed in 36/37 (97.3%) HCWs (Figure 3A). Overall, after the complete vaccination schedule, the whole percentage of full responders (i.e. subjects developing both SARS-CoV-2 specific cellular and humoral response) was 86.5% (32/37). However, 100% of the subjects displayed at least one of the T-cell-mediated cellular response (i.e. either CD4^+^ or CD4^+^ plus CD8^+^) and the humoral response. As expected, none of the 20 HCs enrolled showed SARS-CoV-2-specific T-cell response (supporting information). No significant correlation was observed between age/sex and SARS-CoV-2-specific T-cell response.

### Correlation between SARS-CoV-2-specific neutralizing antibodies and T-cell response

Neutralizing antibody level measured by NTA was soundly correlated with both SARS-CoV-2-specific CD4^+^ (*p* = 0.0001) (Figure 3B) and SARS-CoV-2-specific CD4^+^ plus CD8^+^ (Figure 3C) T-cell response (*p* = 0.0005) at T6. Conversely, no significant correlation was observed between SARS-CoV-2-specific T-cell response and antibody quantification measured by any of the CLIA assays, as representatively depicted for i-Flash assay in Figure 3D and E and in Supplementary figure 1 (Fig. S1) for all the other CLIA tests.

### Neutralizing antibody response against SARS-CoV-2 variants

**Figure 4.**
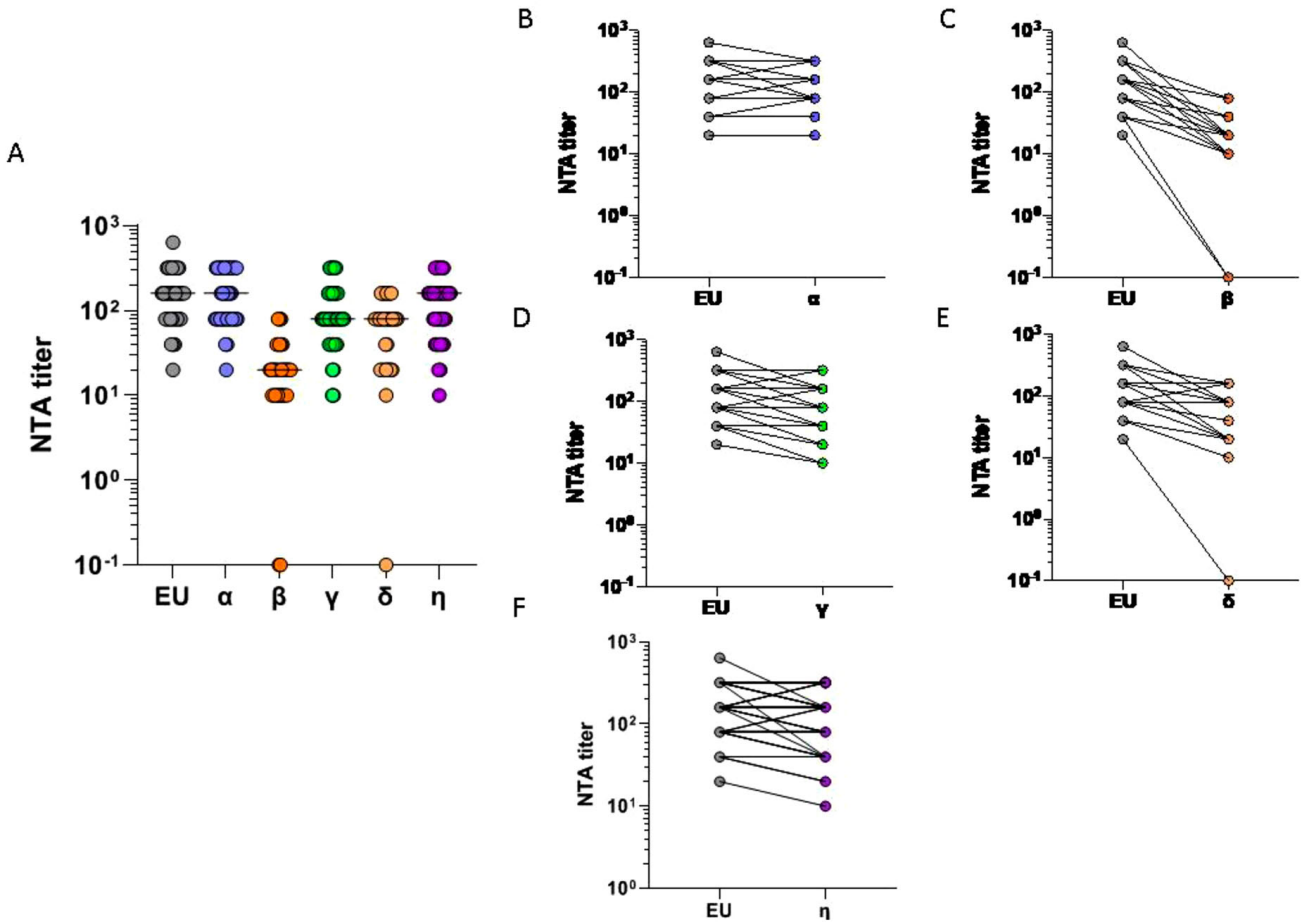
SARS-CoV-2 variants of concern (VOC). Panel (A) Neutralization assay (NTA) performed at T5 on the SARS-CoV-2 lineage B.1 (EU) and 5 VOCs, α-η. Panels (B-F) Comparison between the EU variant and the VOCs α-η, respectively. Lines connect the NTAs of each individual subject.

**Figure 5.**
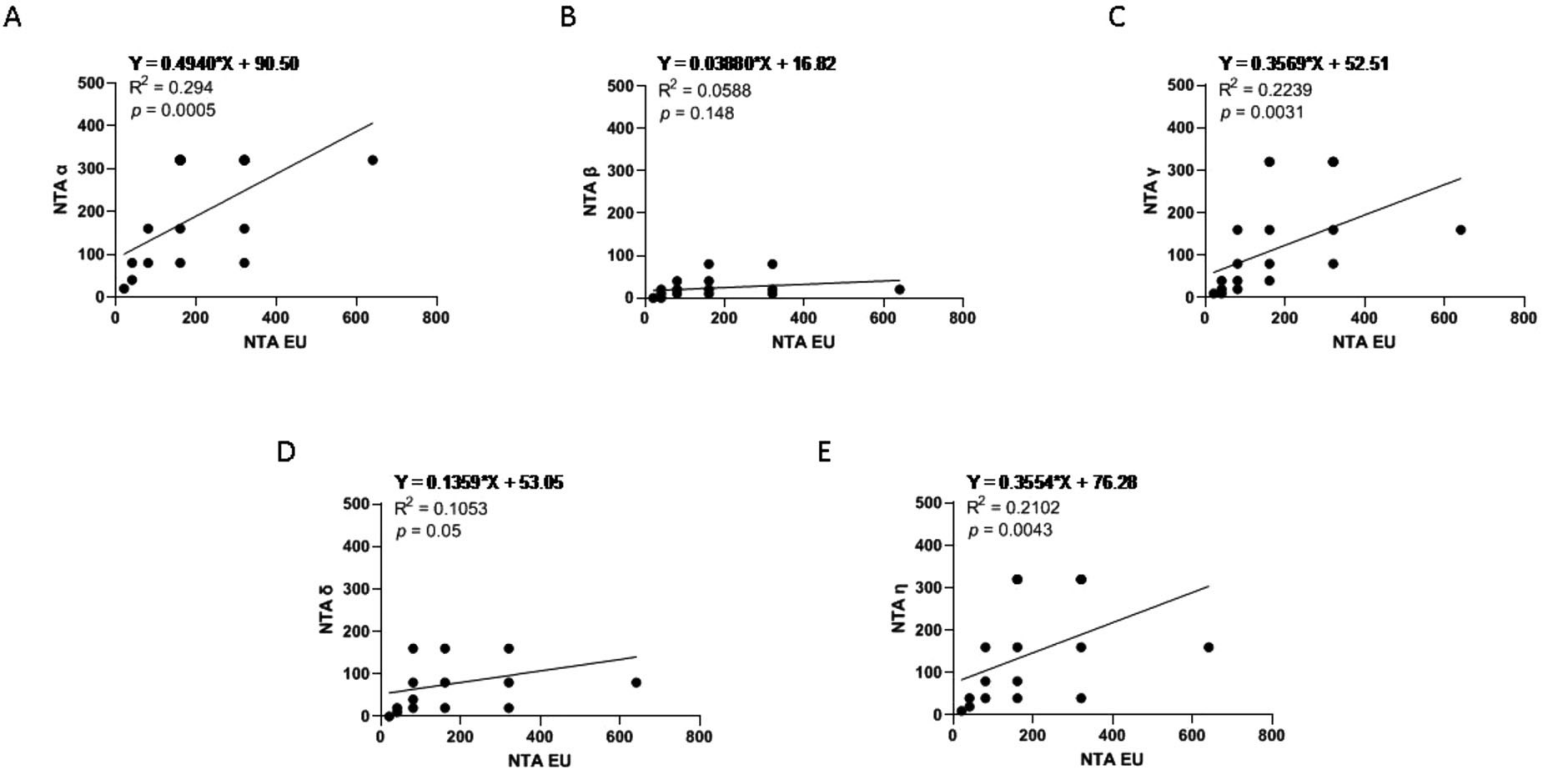
Neutralization assays correlations. Panel (A–E) NTA correlation between the EU variant and the VOCs, respectively, performed at T5. The other correlations between variants are depicted in figure S3.

Sera collected at T5 from all vaccinated HCWs were tested by NTA against the lineage B.1 (EU), assumed as reference virus, as well as Alpha (lineage B.1.1.7), Beta (lineage B.1.351), Gamma (lineage P.1), Delta (lineage B.1.617.2), and Eta (lineage B.1.525) variants (Figure 4(A)). Mean values ± sd were 172.4 ± 124.31 for EU strain (Figure 4), 175.7 ± 113.2 for Alpha (Figure 4(B)), and 114.1 ± 93.76 and 137.6 ± 93.36 for Gamma (Figure 4(D)) and Eta (Figure 4(F)) strains, respectively. Conversely, a reduction of approximatively 55.6% and 86.3% of neutralizing antibody titres was observed from EU to Delta and Beta strains, respectively (Figure 4(E,C)) (mean values ± sd: Delta, 76.5 ± 52.08; Beta, 23.5 ± 19.89). Specifically, there was a 7.3-fold reduction in the neutralization titres against the Beta variant, whereas the fold decline was 2.4 against the Delta strain (Figure 4). Although at lower titres, neutralizing antibodies against the Beta and Delta variants were still detectable in 35/37 (94.5%) and 36/37 (97.2%) subjects, respectively. As a whole, the neutralization ability of Pfizer/BioNTech vaccine immune sera against the EU variant was maintained to that against the Alpha (*p* = 0.0005), Gamma (*p* = 0.0031), and Eta (*p* = 0.0043) variants, but significantly declined in comparison to that against the Beta (*p* = 0.148) and Delta (*p* = 0.051) variants (Figure 5). All the other NTA correlation between variants are reported in Supplementary figure 2 (Fig. S2).

### Correlation between SARS-CoV-2-specific antibodies titre (CLIA) and variant neutralization ability (NTA)

**Figure 6.**
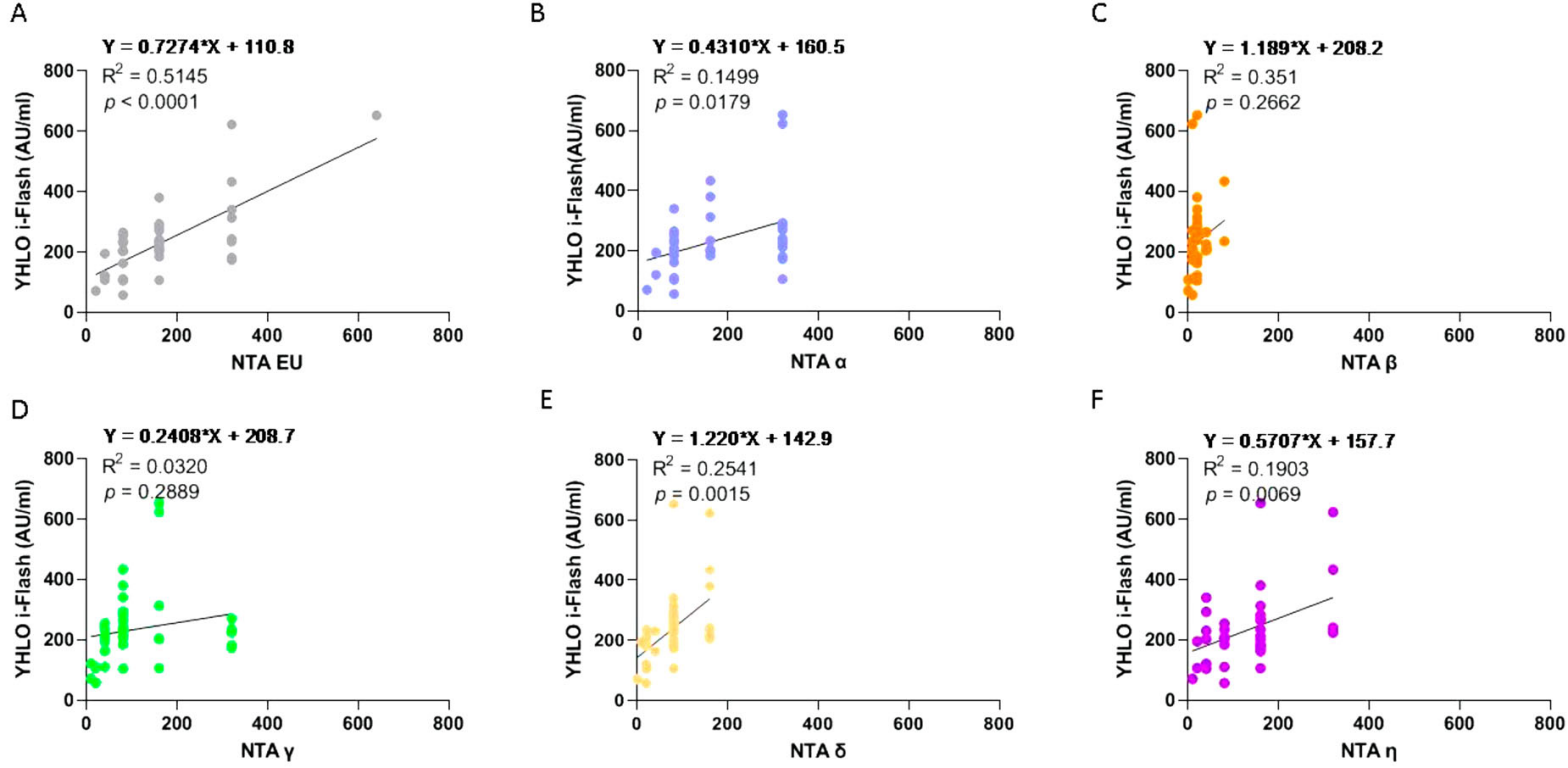
Correlation between anti-SARS-CoV-2 specific antibodies and neutralization assays (NTA). Panel (A–F) correlation between anti-SARS-CoV-2 specific antibodies measured by YHLO i-Flash and the NTA performed on the EU variants and the VOCs, respectively, performed at T5. The correlation with the quantification of anti-SARS-CoV-2 specific antibodies measured by the other methods is depicted in figure S3.

Analyses of correlation between antibody titres measured with different CLIA assays and NTA performed in SARS-CoV-2 variants revealed that none of the CLIA techniques is significantly correlated with the NTA of the whole variants panel, as representatively reported in Figure 6 for i-Flash assay and in supplementary figure 3 (Fig. S3) for all the other CLIA test. On the contrary, NTA of the Delta and the Eta variants significantly correlated with all the CLIA tests employed (Figure 6 and Fig. S3).

## Discussion

In this study, we report a longitudinal prospective and exhaustive estimation of the development of antibody triggered by the mRNA vaccine BNT162b2 for SARS-CoV-2, assessed by the use of different techniques.

Results show that the antibody level was feeble or undetectable after one vaccine dose (T2) in SARS-CoV-2-naive enrolled HCWs, mirroring previous reports investigating the antibody responses in SARS-CoV-2-uninfected subjects after a single dose of SARS-CoV-2 mRNA vaccine [17,18]. Therefore, the administration of only one dose could be not enough to prompt a broad immune defence in uninfected subjects mainly considering that a suboptimal immune response could favour the emergence of viral resistance or escape variants.

Conversely, the administration of two doses of BNT162b2 vaccine elicited a robust SARS-CoV-2-specific immune response, consistently with how previously observed [18], which was detectable both as antibody production and T-cell-driven immune activation in all the enrolled subjects. The antibody titre was measured, at each time point, employing four different CLIA methodologies and tested in a NTA assay to investigate their functional neutralizing properties. Seric antibody titration is the method of choice for determining the SARS-CoV-2 serostatus, on which depends the clinical diagnoses, patients’ management, and determination of seroprevalence within the population for epidemiologic purposes. Overall, results obtained with different techniques were all consistent among one another from a qualitative point of view, confirming that the clinical detection of antibodies targeting the neutralizing viral epitopes reliably represents the actual neutralization capacity [16]. Neutralizing antibodies evolve over time, improving the antigen binding affinity [19]. Consequently, this affinity maturation could explain the apparent discrepancy between the timing of the peak of antibody amount measured by CLIA and the neutralization capability measured by NTA. Moreover, we could not observe a correlation between the quantity of antibody detected by CLIA assays and their neutralizing activity tested by NTA. This may suggest that an NTA, relying on the employment of an actual virus, may elicit a broader antibody array binding sites outside those commonly recognized by commercial CLIA, which could contribute to virus neutralization. On the contrary, the use of completely different cut-off values and quantification ranges of *in vitro* assays and automated tests could be ascribed for the lack of quantitative correlation of the compared assays.

Together with the antibody titre, we evaluated the SARS-CoV-2-specific T-cell response. At T6, an enhanced T-cell activation was detected in all the enrolled subjects compared to uninfected non-vaccinated ones, as shown by QuantiFERON test. The intensity of such immune activation significantly correlated with the neutralization titre assessed by NTA, but oddly, we could not detect a positive correlation with the quantity of antibodies detected by CLIA. The immune response is a complex multifaced process, in which many components partake. Although harder to evaluate in a high-throughput manner, the pivotal role that T-cells cover in SARS-CoV-2-elicited immune response it is now well-established, to the point that it is not uncommon to detect a low antibody titre or neutralization ability associated nonetheless with a protective response able to avoid disease progression [20–23].

In our study, antibody quantification, NTA, and T-cell responses were not affected by age at any time point. However, considering that the natural involution of immune systems occurs nearby 60s [24] and only 3/37 of the enrolled subjects were above this threshold, further studies performed on larger cohort with a wider distribution of ages are necessary to confirm this data. Together with age, a wider cohort would be necessary to confirm gender-related conclusions.

A challenge we are facing at present is the emergence of new SARS-CoV-2 variants that could impair the efficacy of current vaccines as many of the mutations reside in the antigenic supersite in the NTD [25] or in the ACE2-binding site, which is a major target of potent virus-neutralizing antibodies [11,13,26,27]. We tested by neutralization assay many of the variants of concern (VOC), as defined by the CDC [3], together with the EU SARS-CoV-2. To have a deeper insight, we chose not to employ pseudoviruses, but rather the actual authentic isolated. Indeed, the use of lentiviral particles could provide discrepant results compared to natural strains, likely because the mock pseudoviruses cannot completely portray the biology of natural isolates.

Our results indicate that the vaccinated HCWs developed a similar protective NT antibodies response against the EU, Alpha, Gamma, and Eta variants. Conversely, the Beta and the Delta strains displayed the highest immune escape, as demonstrated by the lower neutralization titre. Yet, serum from the majority of vaccinated subjects maintained a neutralizing activity against both Beta (94.5%) and Delta (97.2%) strains that could be active in avoiding the onset of severe COVID-19 symptoms. Unexpectedly, we cannot extrapolate a fully conclusive picture about the correlation between the amount of detected antibodies and the level of protection, as seen by the four CLIA methods employed and the NTA performed on variants, respectively. Indeed, the positive correlation between any of the CLIA employed and the neutralization of the EU, Alpha, Beta, and Delta variants was maintained in almost any case. However, the same could not be assessed for all the Gamma and the Eta variants. Moreover, in this study we did not investigate SARS-CoV-2-specific T-cell response against the different VOC. However, results reported in previous studies suggest that though the efficacy of the antibody response may be somewhat decreased by some mutations naturally acquired by the viral strains, the SARS-CoV-2-specific T-cell response is still preserved, thus granting for protection [23].

In a broader picture, although the Beta variant resulted to have a better immune escape ability, as demonstrated by our NTA assays, it is not the predominant variant right now. Indeed, the Delta variant, although displayed an intermediate immune escape, is by far more concerning, given the higher viral loads, the higher prevalence, and the fast-paced spreading.

These results underline the importance of a solid vaccine-elicited immune response and a robust antibody titre. In turn, this lets us to speculate about the suitability of repeated vaccine boost doses to achieve and maintain the adequate antibody titre overtime. Although further and broader studies should be performed and no firm conclusions can be drawn due to the limited number of analyzed cases, this should be taken into consideration in the definition of vaccinal strategies. Our findings deliver an important message that should not be underestimated. Therefore, we encourage the scientific and medical communities to deeply consider which guidelines should be more appropriate.

## Supplementary Material

BNT162b2_HCW_SUPPLEMENTARY_INFORMATION_EM_I_modified_2.11.2021.doc
