# Supplementary material for: SARS-CoV-2 mRNA vaccine BNT162b2 triggers a consistent cross-variant humoral and cellular response
Source: Emerg Microbes Infect. 2021 Dec 1;10(1):2235–43. doi: 10.1080/22221751.2021.2004866 (PMC8648019; doi:10.1080/22221751.2021.2004866)
Supplement: BNT162b2_HCW_SUPPLEMENTARY_INFORMATION_EM_I_modified_2.11.2021.doc [file TEMI_A_2004866_SM8300.doc]

**SUPPLEMENTARY INFORMATION**

**Neutralization Assay**

Neutralizing antibody titres against SARS-CoV-2 obtained by Neutralization assay (NTA) was defined as follow.

Briefly, 50 μL of each serum, starting from a 1:10 dilution followed by serial two-fold series, were transferred in two wells of 96-weel microtitre plates (COSTAR, Corning Incorporated, NY 14831, USA) and mixed with 50 µL of tissue culture infecting dose 50 (TCID_50_) of SARS-CoV-2. All dilutions were made in DMEM with addition of 1% penicillin and streptomycin. After 1 h incubation at 37 °C and 5% CO_2_, 50 µL of 2x10^4^ Vero E6 (VERO C1008 ATCC® CRL-1586™) cells were added to each well. After 72 h of incubation at 37°C and 5% CO_2_, wells were stained with 0.1% crystal violet solution (Merck KGaA, 64271 Damstadt, Germany) plus 5% formaldehyde 40% m/v (Carlo ErbaSpA, Arese (MI), Italy) for 30 min. Microtiter plates were then washed in running water. Wells were scored to evaluate the degree of cytopatic effect (CPE) compared to the virus control. Blue staining of wells indicated the presence of neutralizing antibodies. Neutralizing titre was the maximum dilution with the reduction of 90% of CPE. A positive titre was equal or greater than 1:10. Positive and negative controls were included in all test run.

Every test included serum control (1:10 dilution), cells control (Vero E6 cells alone) and viral control (three-fold series dilution).


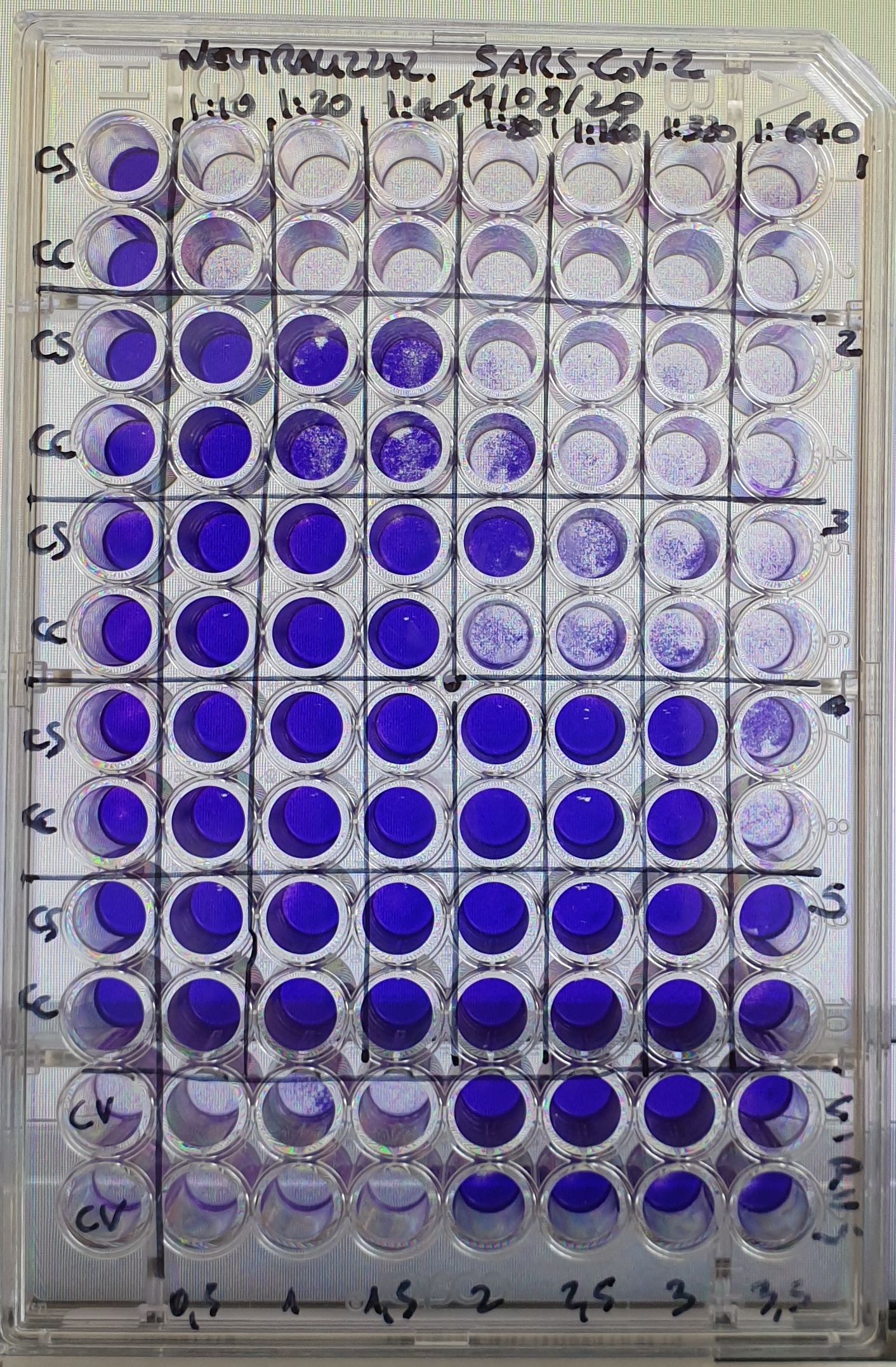


**Figure: A picture of exemplificative neutralization plate**

All the samples are tested in duplicate in two-fold scalar dilution indicated on the top of the plate (1:10, 1:20, 1:40, 1:80, 1:160, 1:320 and 1:640). Blue staining of wells indicated the presence of neutralizing antibodies; CS, serum control; CC, cell control; CV, virus control; 1-digit numbers on the right of the plate are pts identification numbers; 11/08/20 on the top of the plate indicate the assay date (August,11, 2020).

**iFlash SARS-CoV-2 IgG and IgM**

All samples collected at T0 and T5 were analyzed with iFlash SARS-CoV-2 IgG and IgM (Shenzhen YHLO Biotech Co, Shenzhen, China) to exclude a possible ongoing asymptomatic infection since that the assay targets antibodies anti Nucleocapside and Spike proteins (non-neutralizing antibodies). Table 1 reports the assay results.

|  | **One day before vaccination (T0)** | | **Thirty days after II dose (T5)** | |
| --- | --- | --- | --- | --- |
|  | **iFlash SARS-CoV-2 IgG and IgM (YHLO)** | | | |
| **n° subjects** | **Neg < 10 AU/mL Pos > 10 AU/mL** | **Neg < 10 AU/mL Pos > 10 AU/mL** | **Neg < 10 AU/mL Pos > 10 AU/mL** | **Neg < 10 AU/mL Pos > 10 AU/mL** |
|  | **IgG** | **IgM** | **IgG** | **IgM** |
| **1** | 0.1 | 0.2 | 0.2 | 0.5 |
| **2** | 0.2 | 0.3 | 0.6 | 1.2 |
| **3** | 0.1 | 0.2 | 0.5 | 0.5 |
| **4** | 0.1 | 0.2 | 0.2 | 0.4 |
| **5** | 0.1 | 0.5 | 0.3 | 2.2 |
| **6** | 0.1 | 0.2 | 0.5 | 0.6 |
| **7** | 0.1 | 0.2 | 0.2 | 0.6 |
| **8** | 0.1 | 0.2 | 0.4 | 0.6 |
| **9** | 0.1 | 0.2 | 0.3 | 0.4 |
| **10** | 0.1 | 0.1 | 0.2 | 0.5 |
| **11** | 0.2 | 0.2 | 0.7 | 0.4 |
| **12** | 0.2 | 0.2 | 0.3 | 0.6 |
| **13** | 0.1 | 0.1 | 0.4 | 0.4 |
| **14** | 0.1 | 0.1 | 0.2 | 0.4 |
| **15** | 0.1 | 0.1 | 0.4 | 0.4 |
| **16** | 0.1 | 0.2 | 0.3 | 0.5 |
| **17** | 0.1 | 0.2 | 0.2 | 0.7 |
| **18** | 0.1 | 0.2 | 0.3 | 0.5 |
| **19** | 0.1 | 0.2 | 0.3 | 0.5 |
| **20** | 0.1 | 0.2 | 0.3 | 0.5 |
| **21** | 0.3 | 0.7 | 0.2 | 0.6 |
| **22** | 0.1 | 0.2 | 0.2 | 1.2 |
| **23** | 0.4 | 0.7 | 0.5 | 0.7 |
| **24** | 0.5 | 0.6 | 0.5 | 1.0 |
| **25** | 0.1 | 0.2 | 0.2 | 0.8 |
| **26** | 0.2 | 0.2 | 0.3 | 0.4 |
| **27** | 0.2 | 0.2 | 0.3 | 0.3 |
| **28** | 0.1 | 0.1 | 0.5 | 1.7 |
| **29** | 0.1 | 0.2 | 0.4 | 0.8 |
| **30** | 0.8 | 3.9 | 0.3 | 0.4 |
| **31** | 0.3 | 0.4 | 0.3 | 0.4 |
| **32** | 0.1 | 0.2 | 0.3 | 0.5 |
| **33** | 0.1 | 0.2 | 0.6 | 0.5 |
| **34** | 0.2 | 0.4 | 0.3 | 1.5 |
| **35** | 0.1 | 0.3 | 0.7 | 0.8 |
| **36** | 0.2 | 0.2 | 0.3 | 0.4 |
| **37** | 0.1 | 0.2 | 0.3 | 0.4 |

**Table 1: iFlash SARS-CoV-2 IgG and IgM assay results are reported in table. Negative and Positive cut-off is 10 AU/mL for both.**

**QuantiFERON® (QNF) SARS-CoV-2 (QIAGEN, Hilden, Germany)**

To evaluate the CD4^+^ and CD8^+^ T cell stimulation after vaccination we quantified the IFN-γ by means of QuantiFERON® (QNF) SARS-CoV-2.

Twenty unvaccinated healthy controls with no nucleocapside and spike antibodies were used as controls (HCs); the results are reported in table 2.

The T cell response is calculated by subtracting the Nil tube value from the Ag tube value (Ag−Nil). A value of 0.15 IU/ml was used as positive cut-off.

| **QuantiFERON® SARS-CoV-2** | | | |
| --- | --- | --- | --- |
| **n° unvaccinated subjects** | **Target** | **Result (IU/mL)** | **Ag-Nil** |
| **1** | **Nil** | 0.06 |  |
|  | **Ag1** | 0.04 | **-0.02** |
|  | **Ag2** | 0.05 | **-0.01** |
|  | **Mitogen** | > 10 |  |
| **2** | **Nil** | 0.11 |  |
|  | **Ag1** | 0.09 | **-0.02** |
|  | **Ag2** | 0.1 | **-0.01** |
|  | **Mitogen** | 8.89 |  |
| **3** | **Nil** | 0.09 |  |
|  | **Ag1** | 0.07 | **-0.02** |
|  | **Ag2** | 0.06 | **-0.03** |
|  | **Mitogen** | > 10 |  |
| **4** | **Nil** | 0.08 |  |
|  | **Ag1** | 0.06 | **-0.02** |
|  | **Ag2** | 0.07 | **-0.01** |
|  | **Mitogen** | > 10 |  |
| **5** | **Nil** | 0.07 |  |
|  | **Ag1** | 0.04 | **-0.03** |
|  | **Ag2** | 0.08 | **0.01** |
|  | **Mitogen** | > 10 |  |
| **6** | **Nil** | 0.11 |  |
|  | **Ag1** | 0.07 | **-0.04** |
|  | **Ag2** | 0.1 | **-0.01** |
|  | **Mitogen** | > 10 |  |
| **7** | **Nil** | 0.09 |  |
|  | **Ag1** | 0.06 | **-0.03** |
|  | **Ag2** | 0.1 | **0.01** |
|  | **Mitogen** | > 10 |  |
| **8** | **Nil** | 0.1 |  |
|  | **Ag1** | 0.07 | **-0.03** |
|  | **Ag2** | 0.1 | **0** |
|  | **Mitogen** | > 10 |  |
| **9** | **Nil** | 0.08 |  |
|  | **Ag1** | 0.07 | **-0.01** |
|  | **Ag2** | 0.09 | **0.1** |
|  | **Mitogen** | > 10 |  |
| **10** | **Nil** | 0.11 |  |
|  | **Ag1** | 0.07 | **-0.04** |
|  | **Ag2** | 0.1 | **-0.01** |
|  | **Mitogen** | > 10 |  |
| **11** | **Nil** | 0.11 |  |
|  | **Ag1** | 0.06 | **-0.05** |
|  | **Ag2** | 0.1 | **-0.01** |
|  | **Mitogen** | > 10 |  |
| **12** | **Nil** | 0.07 |  |
|  | **Ag1** | 0.05 | **-0.02** |
|  | **Ag2** | 0.1 | **0.03** |
|  | **Mitogen** | > 10 |  |
| **13** | **Nil** | 0.06 |  |
|  | **Ag1** | 0.03 | **-0.03** |
|  | **Ag2** | 0.05 | **-0.01** |
|  | **Mitogen** | > 10 |  |
| **14** | **Nil** | 0.08 |  |
|  | **Ag1** | 0.05 | **-0.03** |
|  | **Ag2** | 0.09 | **0.01** |
|  | **Mitogen** | > 10 |  |
| **15** | **Nil** | 0.1 |  |
|  | **Ag1** | 0.07 | **-0.03** |
|  | **Ag2** | 0.1 | **0** |
|  | **Mitogen** | > 10 |  |
| **16** | **Nil** | 0.07 |  |
|  | **Ag1** | 0.07 | **0** |
|  | **Ag2** | 0.08 | **0.01** |
|  | **Mitogen** | > 10 |  |
| **17** | **Nil** | 0.06 |  |
|  | **Ag1** | 0.04 | **-0.02** |
|  | **Ag2** | 0.08 | **0.02** |
|  | **Mitogen** | > 10 |  |
| **18** | **Nil** | 0.11 |  |
|  | **Ag1** | 0.08 | **-0.03** |
|  | **Ag2** | 0.11 | **0** |
|  | **Mitogen** | > 10 |  |
| **19** | **Nil** | 0.1 |  |
|  | **Ag1** | 0.07 | **-0.03** |
|  | **Ag2** | 0.11 | **0.01** |
|  | **Mitogen** | > 10 |  |
| **20** | **Nil** | 0.08 |  |
|  | **Ag1** | 0.06 | **-0.02** |
|  | **Ag2** | 0.09 | **0.01** |
|  | **Mitogen** | > 10 |  |

**Table 2 : Table shows QuantiFERON® results of twenty unvaccinated healthy subjects**
